# Adjuvant platinum-based chemotherapy in radically resected adrenocortical carcinoma: a cohort study

**DOI:** 10.1038/s41416-021-01513-8

**Published:** 2021-08-16

**Authors:** Otilia Kimpel, Sara Bedrose, Felix Megerle, Alfredo Berruti, Massimo Terzolo, Matthias Kroiss, Knut Mai, Olaf M. Dekkers, Mouhammed Amir Habra, Martin Fassnacht

**Affiliations:** 1 Division of Endocrinology and Diabetes, Department of Medicine, University Hospital, University of Würzburg, Würzburg, Germany; 2 Department of Endocrine Neoplasia and Hormonal Disorders, Unit 1461, The University of Texas MD Anderson Cancer Center, Houston, TX USA; 3 Department of Endocrinology, Diabetes and Metabolism, Baylor College of Medicine, Houston, TX USA; 4 Department of Medical and Surgical Specialties, Radiological Sciences, and Public Health, University of Brescia, Medical Oncology, ASST Spedali Civili, Brescia, Italy; 5 Internal Medicine, Department of Clinical and Biological Sciences, San Luigi Gonzaga Hospital, University of Turin, Orbassano, Italy; 6 Comprehensive Cancer Center Mainfranken, University of Würzburg, Würzburg, Germany; 7 Department of Medicine IV, University Hospital, LMU Munich, München, Germany; 8 Department of Endocrinology & Metabolism, Charité - Charité – Universitätsmedizin Berlin, corporate member of Freie Universität Berlin, Humboldt-Universität zu Berlin, and Berlin Institute of Health, Berlin, Germany; 9 Department of Clinical Epidemiology, Leiden University Medical Center, Leiden, The Netherlands; 10 Department of Endocrinology, Leiden University Medical Center, Leiden, The Netherlands

**Keywords:** Adrenal tumours, Chemotherapy

## Abstract

**Background:**

After radical resection, patients with adrenocortical carcinoma (ACC) frequently experience recurrence and, therefore, effective adjuvant treatment is urgently needed. The aim of the study was to investigate the role of adjuvant platinum-based therapy.

**Methods:**

In this retrospective multicentre cohort study, we identified patients treated with adjuvant platinum-based chemotherapy after radical resection and compared them with patients without adjuvant chemotherapy. Recurrence-free and overall survival (RFS/OS) were investigated in a matched group analysis and by applying a propensity score matching using the full control cohort (*n* = 268). For both approaches, we accounted for immortal time bias.

**Results:**

Of the 31 patients in the platinum cohort (R0 *n* = 25, RX *n* = 4, R1 *n* = 2; ENSAT Stage II *n* = 11, III *n* = 16, IV *n* = 4, median Ki67 30%, mitotane *n* = 28), 14 experienced recurrence compared to 29 of 31 matched controls (median RFS after the landmark at 3 months 17.3 vs. 7.3 months; adjusted HR 0.19 (95% CI 0.09–0.42; *P* < 0.001). Using propensity score matching, the HR for RFS was 0.45 (0.29–0.89, *P* = 0.021) and for OS 0.25 (0.09–0.69; *P* = 0.007).

**Conclusions:**

Our study provides the first evidence that adjuvant platinum-based chemotherapy may be associated with prolonged recurrence-free and overall survival in patients with ACC and a very high risk for recurrence.

## Background

Adrenocortical carcinoma (ACC) is a rare and aggressive disease with limited therapeutic options and a high rate of recurrence even after complete resection [1–5]. Therefore, effective adjuvant treatments are critically needed [6]. Until now, mitotane is the only drug approved for the treatment of advanced ACC and is used also as adjuvant therapy [1, 7–11]. Adjuvant mitotane is not undisputed and some argue that mitotane while acting as adrenolytic agent has low cure rates [12]. There is also uncertainty about the target plasma concentrations of mitotane required to prevent recurrence in this setting [13–15]. Furthermore, all published data on adjuvant mitotane are retrospectively collected, and randomised trials are lacking. The recruitment of the prospective randomised ADIUVO trial (NCT00777244), investigating the efficacy of adjuvant mitotane versus observation only in patients with low-intermediate risk of recurrence is stopped, but the results are still pending. Awaiting the results of the ADIUVO trail, both the comprehensive ESE-ENSAT guidelines 2018 and the new ESMO-EURACAN guidelines 2020 recommend an adjuvant treatment with mitotane in patients who have a high risk of recurrence (i.e., Stage III or IV, R1 or RX resection, or Ki67 >10%) [1, 9]. Nevertheless, the recurrence rate is still about 50% even after mitotane treatment [7].

The available evidence for adjuvant radiotherapy is even more limited compared to mitotane use. Most published reports indicate a reduced risk of local recurrences by an adjuvant radiotherapy, but only few studies suggest that it is also helpful in prolonging overall recurrence-free and overall survival [16–19]. All of these studies are retrospective and hence confer significant selection bias. Therefore, the ESE and ESMO guidelines suggest its use only on an individual basis in patients with R1 or RX resection or in Stage III.

In other solid malignancies, the use of adjuvant cytotoxic chemotherapy is known to reduce recurrence risk. However, the role of adjuvant chemotherapy in ACC has not been established, and the available evidence is extremely limited [20]. Hovi et al. explored the combination of cisplatin and etoposide in the adjuvant setting in a small series of five ACC patients aged 1 to 21 years [21]. Chemotherapy was given shortly after surgical resection, and all patients remained in complete remission 29 to 109 months later [21]. Another study from Khan et al. tested the combination of streptozotocin plus mitotane as adjuvant therapy in a Phase II trial of 17 patients after complete tumour resection. This study suggests a longer disease-free survival compared with a control cohort of 11 patients, who received no adjuvant therapy (49 vs. 12 months) [22]. However, confounding is likely an issue and it is also not clear if the presumed advantage of adjuvant treatment can be attributed to mitotane, streptozotocin or the combination of both. In line with the limited evidence, ESE and ESMO guideline panelists could not reach a consensus on the use of adjuvant cytotoxic chemotherapy [1, 9]. Both guidelines suggest to consider treatment with an adjuvant platinum-based chemotherapy in selected patients with very high risk for recurrence on an individual basis (e.g. Ki67 >30%. large tumour thrombus in the vena cava, Stage IV or R1 resection). In patients with locally advanced or metastatic ACC, the randomised FIRM-ACT trial demonstrated that the combination of etoposide, doxorubicin, cisplatin and mitotane (EDP-M) was superior to streptozotocin and mitotane [23]. Although the primary endpoint, overall survival, failed (potentially due to the crossover design), EDP-M led to a higher objective response rate (23% vs. 9%) and improved progressive-free survival (5.0 vs. 2.1 months) [23]. So far, no other regimen tested in larger studies could reach similar results [24, 25].

Here, we present the first retrospective study to explore the efficacy and safety of adjuvant platinum-based chemotherapy in adult patients with macroscopically radical resected ACC.

## Subjects and methods

### Study population

This cohort study was part of the ENSAT registry study (www.ensat.org/registry) in four European reference centres for ACC (Würzburg, Germany; Brescia, Italy; Berlin, Germany; and Orbassano, Italy) and the MD Anderson Cancer Center in Houston, US. It was approved by the ethics committees/institutional review boards at all participating institutions and all patients provided written informed consent.

Only patients who had undergone radical surgery between 2002 and February 2020 were included. The follow-up for this study was closed in August 2020. Histological and clinical parameters (sex, age at diagnosis, tumour size, evidence of hormonal excess, tumour stage according to ENSAT [26] classification, date of surgery, Weiss score, Ki67 index, size and number of tumoural lesions, date of starting mitotane, date of starting chemotherapy, mitotane plasma concentration and follow-up information) were retrieved from the ENSAT ACC registry, patients’ histories and medical records. All histological diagnoses were confirmed by experienced pathologists. Tumour staging at diagnosis was based on imaging studies and on the findings during surgery. Patients with macroscopically incomplete resection (either R2 resection or distant metastases that were not removed), lack of relevant information on the primary diagnosis or follow-up, concomitant anti-tumour treatment apart from mitotane (e.g. radiotherapy or other drugs than platinum-based therapies) or the start of adjuvant chemotherapy later than 3 months after surgery were excluded.

Medical records were reviewed for adverse events associated with adjuvant platinum-based chemotherapy. All adverse events were scored according to the National Cancer Institute Common Terminology Criteria Adverse Events (NCI-CTCAE) classification version 5.0 [27].

### Platinum-based chemotherapy and control group

The platinum-based chemotherapy group included patients who met the following predefined criteria: macroscopically radical resected ACC (defined as no evidence of macroscopic residual disease based on surgical reports, histopathological analysis, and postoperative imaging) with resection status R0, Rx or R1, and the start of an adjuvant platinum-based chemotherapy <3 months after primary surgery. Adjuvant platinum-based chemotherapy was defined as monotherapy with cisplatin or carboplatin or in combination with other cytotoxic drugs.

The inclusion criteria for the control group were identical except for the use of platinum-based chemotherapy.

We performed two different methodological approaches for analysis. First, every patient was matched with one control patient according to the following criteria: Ki67 index (+/− 5% in tumours with Ki67 <20%, +/−15% in tumours with Ki67 20-49% and +/−20% in tumours with Ki67 ≥50%) resection status (R0, R1, Rx), tumour stage, concomitant treatment with mitotane (yes/no) and presence of preoperative glucocorticoid excess (yes/no). Matching was performed by an investigator who was not aware of patient outcome. This was done in a hand-picked manner only with the above-mentioned clinical data available for all patients. To reduce the impact of potential immortal time bias, we performed a landmark analysis excluding all patients who experienced recurrent disease or died within 12 weeks after radical resection. Second, we applied a propensity score approach; firstly, we calculated a propensity score for every patient (see below). Subsequently, this propensity score was used in a multivariable model (see below).

### Outcome assessment

Upfront, we defined recurrence-free survival (RFS) as the most relevant outcome for the present analysis. Disease recurrence was defined as unequivocal radiologic evidence of local recurrence and/or distant metastasis during follow-up. Radiological evaluation was performed according to local standards every 2–5 months.

### Statistical analysis

Recurrence-free survival (RFS) was defined as the time from the date of surgery to the first evidence of recurrent disease or last follow-up or death whichever occurs first. Overall survival (OS) was defined as the time from the date of surgery to the date of death or last follow-up. Patients without recurrence or death were censored at the date of last follow-up. Survival analysis was performed using the Kaplan–Meier method, and differences between groups were assessed by log-rank statistics.

In a multivariable approach using the Cox proportional hazards model, recurrence-free and overall survival were adjusted for the following variables: resection status, tumour stage, presence of glucocorticoid excess, Ki67 index and adjuvant mitotane therapy

Secondly, we performed a propensity-matched analysis. Using logistic regression, we estimated a propensity score for every patient based on the following prognostic variables: age at diagnosis, sex, tumour size, ENSAT stage, Ki67category, glucocorticoid excess and adjuvant mitotane. Subsequently, the multivariable Cox analysis included the propensity score.

To avoid immortal time bias a time-dependent approach was chosen for both methods [28], using chemotherapy as a time-dependent variable. Here, only the person-time at risk (not including the time until the start of chemotherapy) was counted.

Data were analysed using SPSS v.26 (IBM SPSS Statistics) and STATA 16.0.

## Results

### Patient characteristics

The total cohort consisted of 299 patients and key patients’ characteristics are given in Table 1. Thirty-one of them were treated with adjuvant platinum-based chemotherapy. In comparison to the entire control group, the median Ki67 index was higher (30% vs. 20%, *P* = 0.008), more patients had ENSAT tumour Stages III and IV, and more patients were treated with adjuvant mitotane in the platinum-based chemotherapy group. The control group included more women, with higher age, less patients with glucocorticoid excess and R0 resection, and the median tumour diameter was slightly smaller (Table 1).

**Table 1:** Baseline characteristics of the patients.

	Adjuvant platin therapy (*n* = 31)	Matched controls (*n* = 31)	*P* value platin vs matched controls	Entire control cohort (*n* = 268)	*P* value platin vs entire control group
Sex (F:M)	16:15	19:12	0.44	177:91	0.11
Median age yrs (range)	41 (4–59)	44 (18–67)	0.79	49 (4–79)	0.066
Median tumour size mm (range)	124 (25–300)	120 (38–220)	0.79	110 (25–260)	0.45
Autonomous hormone secretion					
Cortisol +/− androgens− *n* (%)	15 (48.4)	12 (38.7)	0.068	101 (37.7)	0.11
Androgens	5 (16.1)	3 (9.7)		22 (8.2)	
Aldosterone	0	1 (3.2)		6 (2.2)	
Estrogens	0	0		0	
Inactive	7 (22.6)	15 (48.4)		119 (44.4)	
Unknown	4 (12.9)	0		20 (7.5)	
ENSAT tumour stage					
I, *n* (%)	0	0	1.0	14 (5.3)	0.026
II, *n* (%)	11 (35.5)	11 (35.5)		138 (52.2)	
III, *n* (%)	16 (51.6)	16 (51.6)		101 (38.4)	
IV, *n* (%)	4 (12.9)	4 (12.9)		10 (3.8)	
Venous tumour thrombus^a^, *n* (%)	10 (32.3)	10 (32.3)	1.0	16 (6.3)	<0.001
Resection status					
R0, *n* (%)	25 (80.6)	25 (80.6)	1.0	183 (68.3)	0.56
RX, *n* (%)	4 (13)	4 (13)		54 (20.1)	
R1, *n* (%)	2 (6.4)	2 (6.4)		30 (11.2)	
Ki67 index—median (range)	30 (10-80)	32.1 (8-80)	0.86	20 (1–90)	0.008
<20%	7 (25)	5 (17.9)	0.55	92 (44.7)	0.014
20–39%	10 (35.7)	14 (50)		79 (38.3)	
≥40%	11 (39.3)	9 (32.1)		35 (17.0)	
Number of patients with adjuvant mitotane (%)	28 (90.3)	28 (90.3)	1.0	120 (44.9)	<0.001
Highest mitotane plasma concentration (mg/L)—median (range)					
In the first 3 months	12 (3–28)	10 (1–23)	0.87		
No. of analysed patients	*n* = 20	*n* = 23			
Until progress/end of therapy	18 (3–34)	17 (1–27)	0.86		
No. of analysed pts.	*n* = 24	*n* = 24			
No. of patients with mitotane level >14 mg/L during therapy (%)	17 (54.8)	17 (54.8)	1.0		

^a^In the inferior vena cava or renal vein.

### Platinum-based chemotherapy

The majority of patients was treated with a combination of either cisplatin or carboplatin plus etoposide (for details see Table 2). In median, treatment had started 38.5 days (13–71) after surgery and four cycles [2–8] of chemotherapy have been administered. Twenty-eight of 31 patients have been treated concomitantly with adjuvant mitotane, and plasma mitotane levels were almost identical to the matched control group (Table 1). Using a multivariate analysis, there was no significant difference in recurrence-free survival, although patients treated with cisplatin (*n* = 21) seemed to do better than with carboplatin (*n* = 10) (HR = 0.26, 95% CI 0.03–2.43; *P* = 0.24). Neither a significant difference in recurrence-free survival was detectable if 2–3 cycles (*n* = 8) or four and more cycles (*n* = 23) have been applied (HR = 0.47, 95% CI 0.10–2.1; *P* = 32).

**Table 2:** Details on platinum-based chemotherapy and the number of cycles administered.

Chemotherapy regimen, *n* (%)	Number of patients (%)	Median number of cycles (min–max)
Cisplatin/etoposide (d1-3 100 mg/m^2^ E, d2-3 40 mg/m^2^ P; every 3–4 weeks)	16 (51.6)	4 (2–8)
Carboplatin/etoposide (d1-3 100 mg/m^2^ E, d3 P AUC 5; every 3–4 weeks)	8 (25.8)	4 (2–6)
Cisplatin/etoposide/doxorubicin (d1 40 mg/m^2^ D, d2-4 100 mg/m^2^ E, d3-4 40 mg/m^2^ P; every 4 weeks)	5 (16.2)	4 (3–6)
Carboplatin/etoposide/doxorubicin (d1 40 mg/m^2^ D, d2-4 100 mg/m^2^ E, d4 P AUC 5; every 4 weeks)	1 (3.2)	4 (3–4)
Cisplatin	1 (3.2)	2

*d* day, *E* etoposide, *P* cisplatin and carboplatin, respectively, *D* doxorubicin.

### Clinical outcomes using the matched control cohort

Tumour response was assessed similarly between groups: thoracic and abdominal computed tomography (*n* = 17 in the platinum group vs. *n* = 20 in the control group), thoracic computed tomography and abdominal magnetic resonance imaging (*n* = 5 vs. *n* = 7 or FDG-PET/CT (*n* = 9 vs. *n* = 4). There was no significant difference in the time intervals for imaging between the groups (platinum-based group 3.2 ± 1.6 months vs. 3.7 ± 2.2 months in the control group for the first imaging and platinum-based group 6.0 ± 2.0 months vs. 8.0 ± 3.0 months in the control group for the second imaging). The median time of follow-up in the platinum group was 27.1 (3.0–182.0) months and in the control group 37.4 (3.1–133.1) months.

**Fig. 1:**
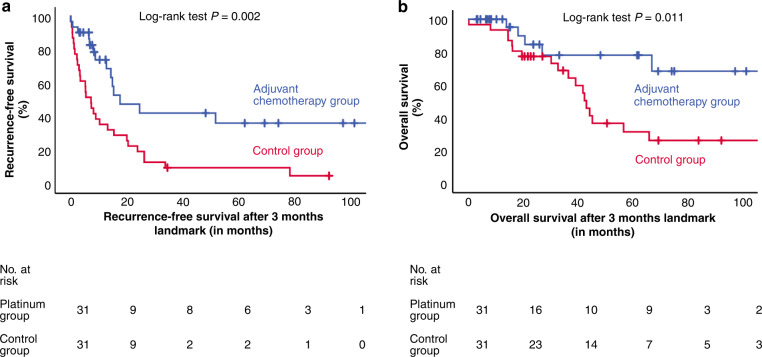
Recurrence-free and overall survival in patients with ACC and very high risk of recurrence. Kaplan–Meier estimates of recurrence-free survival (**a**) and overall survival (**b**) applying a landmark analysis 3 months after surgery in 31 patients with ACC treated with platinum-based chemotherapy and 31 matched controls. Adjusted HR for PFS from Cox analysis is 0.19 (95% CI 0.09–0.42; *P* < 0.001) and for OS 0.26 (95% CI 0.09–0.72; *P* = 0.010).

Fourteen of 31 patients with adjuvant platinum-based therapy experienced recurrence, whereas this was the case in 29 of 31 matched controls. Patients with adjuvant platinum-based therapy had a longer median RFS than matched controls (20.5 months vs. 9.1 months; *P* < 0.001; Fig. 1a). In a multivariable analysis, the adjusted hazard ratio (HR) for RFS was of 0.35 (95% CI 0.19–0.67; *P* = 0.001). Applying a landmark approach, median RFS three months after surgery was 17.7 vs. 7.3 months; *P* = 0.002) leading to an adjusted HR of 0.19 (95% CI 0.09–0.42; *P* < 0.001). Using a time-dependent exposure analysis, the 14 recurrences in the chemotherapy group occurred in 896.7 person-months, whereas the 29 recurrences in the control group occurred in 573.7 person-months yielding a relative risk reduction of 0.32.

Median overall survival after the landmark of 3 months was not reached in the adjuvant chemotherapy group and was 43.1 months in the control group. At last follow-up, 5 patients in the chemotherapy group and 19 patients in the control group had died; there were no deaths unrelated to ACC. Overall survival was longer in the platinum-treated group (adjusted HR 0.26; 95% CI 0.09–0.72; *P* = 0.010; Fig. 1b).

There was no difference regarding the pattern of recurrence in the platinum group and the matched control group.

### Clinical outcome using propensity score matching

In addition to the matched control analysis, we performed a second approach with propensity score matching. After adjustment for propensity scores and accounting for immortal time bias, the HR for RFS was 0.45, 95% CI 0.29–0.89, *P* = 0.021. The HR for OS was 0.25 (95% CI 0.09–0.69; *P* = 0.007), respectively.

### Adverse events in patients with platinum-based chemotherapy

The documented adverse events associated with platinum-based chemotherapy were all well-known and mostly mild or moderate (Table 3). Neither Grade 4 nor Grade 5 events occurred. Only in one patient, a Grade 3 event with febrile neutropenia and oral mucositis was recorded. All patients showed a decrease of neutrophil cells, but only in the above-mentioned patient clinical sequels developed. Most of the patients suffered from vomiting, nausea and fatigue Grades 1 and 2. All patients experienced alopecia. No patient suffered from heart, hepatic or renal failure or nervous system disorders.

**Table 3:** Adverse events according to the NCI CTC criteria v5.0 (27).

Adverse event	Grade 1	Grade 2	Grade 3
Anaemia	8	0	0
Neutrophil count decreased	24	7	1
Febrile neutropenia			1
Ear and labyrinth disorders	1	0	0
Mucositis oral	0	0	1
Vomiting	5	3	0
Nausea	16	6	0
Fatigue	8	4	
Alopecia	0	31	
Weight loss	11	2	0
Peripheral neuropathy	0	0	0

Neither Grade 4 nor Grade 5 events occurred.

## Discussion

In this report, we present the first cohort study of adult patients with ACC treated with adjuvant platinum-based therapy. The aim of our analysis was to provide exploratory evidence for or against the use of this potentially toxic therapy in patients with a very high risk of recurrence. The results of this study were clearly in favour of adjuvant platinum therapy. To ascertain the efficacy of adjuvant chemotherapy, we performed two statistical approaches. First, we used well-matched controls (accounting for the key prognostic factors like ENSAT stage, resection status, Ki67 index, cortisol excess, but also the use of concomitant mitotane treatment). Second, we performed a propensity score matching using the entire cohort of 299 patients. Both approaches clearly suggest that patients treated with an adjuvant platinum-based chemotherapy have a significantly decreased risk of recurrence. Twenty-nine of 31 patients (94%) in the matched control group experienced recurrence, whereas this was the case in only 14 of 31 (45%) of the platinum-based therapy group. Furthermore, these results were confirmed when we applied two different analyses to account for an immortal time bias, namely a landmark approach and a time-dependent exposure analysis. The very high recurrence rate in the control group—despite the fact that more than 90% of patients were treated with adjuvant mitotane—confirmed the very high-risk constellation identified by the above-mentioned prognostic factors. Overall, adjuvant platinum-based therapy was associated with a risk reduction in recurrence of ~65%. Furthermore, this effect seems to translate also to a significantly improved overall survival with a risk reduction for mortality of ~70%, respectively.

Our study has obvious limitations including the retrospective nature and lack of randomisation in addition to the relatively small sample size. However, due to the virtually absent evidence for the application of cytotoxic chemotherapy in an adjuvant setting in ACC and the consecutive lack of a clear recommendation for its use, it is unlikely that a larger cohort will be recruited in the near future. Furthermore, to each patient in the ‘platinum group’ only one control patient could be matched. Other limitations are the various platinum-based chemotherapy regimens and the different combinations of drugs and number of cycles and the non-standardised treatment with mitotane. As expected for a group of high-risk recurrence patients, almost all patients in the ‘platinum group’ have been treated with mitotane. However, the same number of patients were treated with mitotane in the matched controls and the documented mitotane plasma level was similar.

In addition, we have to acknowledge that the decision for (or against) adjuvant platinum-based chemotherapy was made by local staff and was not based on any defined criteria. However, it is obvious that these patients had a perceived very high risk of recurrence. Nevertheless, the results cannot be generalised.

We are well aware that our study only provides first evidence supporting the use of adjuvant platinum-based therapy in ACC. However, it clearly underlines the need for a randomised trial on this topic to eliminate the uncertainties and limitations of retrospective cohort studies. Recently, an international consortium initiated such a trial which reflects an excellent opportunity to include ACC patients with a very high risk of recurrence (NCT03583710, NCT03723941). We certainly have to acknowledge that there is no universally accepted definition of presumably very high-risk patients. However, our study provides some hint that the suggestion by the ESE-ENSAT guidelines in this context seems to be reasonable. In these guidelines, the panelists propose with caution that in patients with one of the following risk factors Ki67 >30%, large tumour thrombus in the vena cava, Stage IV, or R1 resection, adjuvant chemotherapy should be considered [9]. Furthermore, in some selected patients (e.g. after R1 resection) even a combination of mitotane plus etoposide and cisplatin with local radiotherapy could be considered. However, data on this combination are completely lacking.

In summary, our study indicates that adjuvant treatment with platinum-based chemotherapy is associated with beneficial effects on clinical outcome in patients with adrenocortical carcinoma with a very high risk of recurrence. We believe that our retrospective analysis should raise interest in adjuvant chemotherapy as a treatment tool for this disease in selected patients. In the future, prospective, randomised trials like ADIUVO-2 will finally define the role of an adjuvant platinum-based chemotherapy in adrenocortical carcinoma.

### Reporting summary

Further information on research design is available in the Nature Research Reporting Summary linked to this article.

## Supplementary information

Reporting Summary

## Data Availability

The datasets generated and/or analysed during this study are not publicly available due to privacy issues of the patients with a very rare disease but are available in an anonymized fashion from the corresponding author on reasonable request.
